# How Really Ancient Is Paulinella Chromatophora?

**DOI:** 10.1371/currents.tol.e68a099364bb1a1e129a17b4e06b0c6b

**Published:** 2016-03-15

**Authors:** Luis Delaye, Cecilio Valadez-Cano, Bernardo Pérez-Zamorano

**Affiliations:** CINVESTAV Irapuato; Ingeniería Genética, CINVESTAV - Irapuato; Irapuato, Guanajuato, Mexico

## Abstract

The ancestor of Paulinella chromatophora established a symbiotic relationship with cyanobacteria related to the Prochloroccocus/Synechococcus clade. This event has been described as a second primary endosymbiosis leading to a plastid in the making. Based on the rate of pseudogene disintegration in the endosymbiotic bacteria Buchnera aphidicola, it was suggested that the chromatophore in P. chromatophora has a minimum age of ~60 Myr. Here we revisit this estimation by using a lognormal relaxed molecular clock on the 18S rRNA of P. chromatophora. Our time estimates show that depending on the assumptions made to calibrate the molecular clock, P. chromatophora diverged from heterotrophic Paulinella spp. ~ 90 to 140 Myr ago, thus establishing a maximum date for the origin of the chromatophore.

## Introduction

Mitochondria and plastids evolved from free­living bacteria by symbiogenesis more than one billion years ago^1^. Both events boosted the evolution of eukaryotes by expanding their metabolic abilities. Primary endosymbiosis leading to organelles (i.e., mitochondria and plastids) was thought to be unique in the history of life until the recent discovery of an independent primary endosymbiosis in *Paulinella chromatophora* 2. This thecate filose amoeba hosts in its cytoplasm photosynthetic organelles of cyanobacterial origin, called chromatophores. Phylogenetic analysis of 16S rRNA showed that the chromatophores originated from marine α­cyanobacteria from the *Prochlorococcus*/*Synechococcus* clade. In contrast, “classical” plastids of plants and algae evolved from an ancient unknown lineage of cyanobacteria^3^.

The genome of the cyanobacteria *Synechococcus* WH5701, one of the closest known free­-living relatives of the chromatophore with a sequenced genome, has 2917 protein­-coding genes^4^. In contrast, the chromatophores of *P*. *chromatophora* strains FK01 and M0880/a contain between 841 and 867 protein­-coding genes respectively^5^^,^ 6. Remaining genes in the chromatophore suggest a strong metabolic interdependence with the amoebal nucleocytoplasm. The discovery that proteins of the chromatophore photosynthetic apparatus are encoded in the host genome and imported back into the cyanobacterial­-derived compartment, reinforces the suggestion that the chromatophore is a bona fide primary organelle^7^.

It is likely that the origin of the chromatophore is one or two orders of magnitude more recent than the establishment of the primary plastids of plants and algae. But, how ancient is the chromatophore? Since there is no fossil record of *P*. *chromatophora* or its close relatives, it is difficult to have a precise answer to this question. However, an initial guess suggested the chromatophore has a minimum age of 60 Myr. This was based on the proposal that in *Buchnera* *aphidicola* (Aphid’s endosymbiotic bacteria) a pseudogene needs between 40-60 Myr to disintegrate completely^8^. Since the genome of the chromatophore has pseudogenes, the same tempo was extrapolated for the chromatophore^6^.

Surprisingly, the suggestion that the chromatophore in *P*. *chromatophora* has a minimum age of 60 Myr underwent a change in part of the subsequent literature indicating that *P*. *chromatophora* has ~60 Myr. Clearly, this is an assertion that requires clarification and further analysis. The recent identification by single cell genomics of non-­photosynthetic close relatives of *P*. *chromatophora* 9 and novel attempts to calibrate the origin of major eukaryotic groups^10^^,^ 11 offer an opportunity to revisit the origin in time of this extraordinary symbiosis.

## Methods

Taxon sampling. Based on previous published phylogenetic analyses we retrieved a sample of 18S rRNA sequences from rhizaria and stramenopila from SILVA database^12^. These included sequences from: i) the phylogenetic analysis whereby close relatives of *P*. *cromatophora* were identified^9^; ii) the phylogenetic analyses describing divergence times of major eukaryotic groups^10^^,^ 11; and iii) sequences from phylogenetic analyses of euglyphids^13^ and diatoms^14^. We did not include sequences from foraminifera due to their high rate of evolution reflected in extreme long branches. Sequences were aligned with MUSCLE^15^. The final alignment contained 43 sequences and 1128 aligned positions without gaps. The phylogenetic tree reconstructed from these sequences (see below) is in general terms congruent with published phylogenetic analyses.

Molecular clock analyses. BEAUTi was used to prepare xml files. Time trees were inferred by using a lognormal relaxed molecular clock as implemented in BEAST 2^16^. To select the model of evolution we used jModelTest^17^. The Tamura and Nei 1993 model (TrN) plus gamma distribution and an estimated proportion of invariant sites were rated best by the bayesian information criterion (BIC). Therefore, all the analyses were conducted under this model of evolution. In addition, we used a Yule tree prior for all analyses.

To calibrate the clock, we relied upon several sources of information (Fig 2). These included organismal as well as chemical fossils. We also used previous published estimates of the time divergence between rhizaria and stramenoplia. These sources of information were organized into four different calibration schemes. All four schemes used information from non­-controversial fossil record (evidences: a, b, c and d). However, the schemes differ on the use of soft evidence from previous molecular clock analyses (evidence: e.1 and e.2); and on the use of evidence from vase­shaped microfossils (VSM) described by^18^. VSM were originally assigned to rhizaria. However, a posterior molecular clock analysis favored a re­interpretation of VSM as members of amoebozoa^10^. Thus, the four calibration schemes were: (a, b, c, d, e.1); (a, b, c, d, e.1, f); (a, b, c, d, e.2); (a, b, c, d).

Priors on the age of nodes were adjusted to reflect confidence on time divergence. For instance, we assigned a strong prior on the origin of rhizosolenid diatoms because the origin of this lineage has been dated to 91.5 ± 1.5 Myr based on chemical fossils^14^. The 95% probability distribution on this case is between 91.5 to 97.0 Myr. Other calibrations received less strong priors, as indicated by the larger number of years contained along the 2.5% to 9.5% quantiles of the shape column from Table 1. Priors used for the origin of pennate diatoms and for the origins of earliest diatoms were based on those used by^11^ and represent minimal divergence times. The prior used for the origin of Eugliphydae was based on the supposition that the group is much older than the fossil evidence as suggested by^19^. This prior also represents a minimal divergence time. Finally, to assign a prior to the divergence in time of rhizaria from stramenopila we first averaged BEAST time estimates published by^11^ and then we parametrized a normal distribution to include the variability of these estimates. We used this normal distribution as a prior. We constructed a second prior for the same divergence of rhizaria from stramenopila but now based on the inference published by^10^. All sequences within each prior were restricted to be monophyletic. The root of the tree was determined by making rhizaria monophyletic.

We evaluated each calibration scheme by looking at convergence and ESS > 200 in Tracer after running chains of length 10^10^ and sampling each 10^4^ generations. We discarded 10% of generations as burnin. Finally, we conducted all analyses without sequences (i.e., sampling from priors) to determine the impact of priors and to test whether sequences are informative on estimated divergence dates. Trees were obtained with TreeAnnotator and visualised with FigTree. We provide BEAUTi xml files of the four calibration schemes for BEAST 2 analyses here.

## Results and discussion

In Fig. 1 we show an estimate of the divergence in time of *P*. *chromatophora* by using a lognormal relaxed clock on 18S rRNA. To calibrate this tree we relied on: a) the origin of rhizosolenid diatoms, which is known with high confidence (91.5 ± 1.5 Myr)^14^; b) a minimal time divergence of pennate diatoms (80 Myr)^11^^,^ 20; c) a minimal time divergence for diatoms (133.9 Myr)^11^^,^ 21; d) a minimal time divergence of Euglyphidae 40 Myr^19^; and e.1) a time estimation of the divergence of rhizaria from stramenopila ~1232 Myr^11^. This gave us an estimation for the origin of *P*. *chromatophora* of 93.6 Myr (38.1 – 138.2).

**The origin of P. chromatophora in time according to SSU rRNA lognormal molecular clock.**
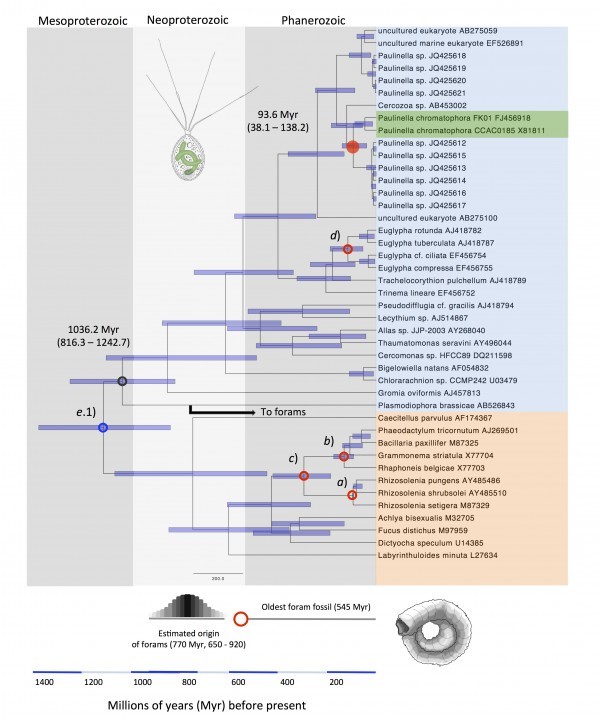
Tree calibrated under the scheme (a, b, c, d, e.1). Small open red circles indicate calibrations based on fossil record (a, b, c, and d); small open blue circle indicate calibration based on previous estimation (e.1); red full circle, estimated age of *P*. *chromatophora*. Notice that the divergence in time of *Plasmodiophora* *brassicae* (black open circle) is consistent with the proposed origin in time of forams. Species names in orange: stramenopila; species name in blue and green: rhizaria.

**Calibration constrains.**
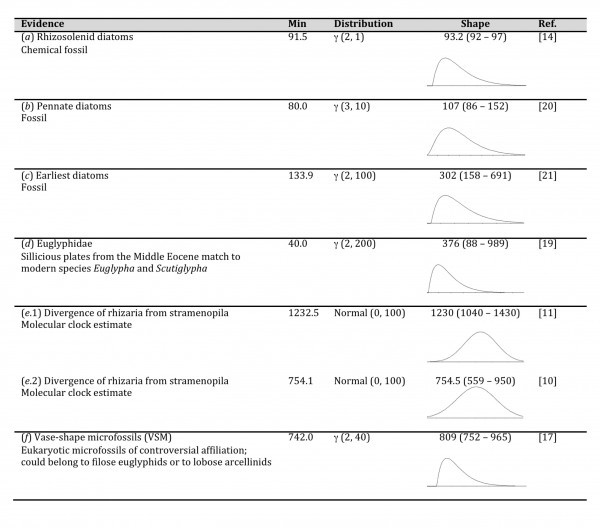
Evidence: used to derive priors for calibration constraints; Min: Minimal divergence time (Offset parameter in BEAUTi) in Myr; Distribution: Used to model each prior together with their respective parameter values (alpha α and beta β for gamma γ and mean and sigma σ for normal distributions); Shape: shows the median for each distribution and the values containing the 2.5% and 97.5% quantiles. Stronger priors span few years between the 2.5% and 97.5% quantiles.

However other calibration schemes are possible. For instance, we can use the report of vase­-shaped microfossils (VSM) of ~742­ to 770 Myr (calibration f) that were described tentatively as members of Euglyphida^18^ together with calibrations a, b, c, d and e.1, to get an estimate of 141.4 Myr (48.7 – 210.4) for the origin of *P*. *chromatophora*. Alternatively, we can follow the suggestions that VSMs belong to amoebozoa and that rhizaria diverged from stramenopila 754.1 Myr (639.8 – 903.3)^10^ together with calibration points a, b, c, d and e.2 to get an estimate of 55.8 Myr (25.4 – 78.6) for the origin of *P*. *chromatophora*. Finally, we can estimate the divergence of *P*. *chromatophora* based only on calibration points a, b, c and d; thus avoiding controversial VSM fossils (calibration f) and the soft information provided from previous estimates of the divergence between rhizaria and stramenopila (calibrations e.1 and e.2). By this, we get an estimate of 47.9 Myr (28.8 – 64.9).

Which of the four time estimates is closer to the true divergence time of *P*. *chromatophora*? Foraminifera offer a clue. Foraminifera belong to rhizaria and as well as diatoms have a detailed fossil record. However, their 18S sequences are too divergent to include them in our analysis. The first appearance of forams in the fossil record is dated at 545 Myr^22^. Recent molecular time estimates of forams suggest this group originated 770 Myr (650 – 920)^23^. A phylogenetic analysis based on 15 concatenated genes shows that forams are a sister group of *Plasmodiophora* *brassicae* with the exclusion of *Gromia* *oviformis* as an outgroup^11^. Therefore, the origin of the lineage leading to *P*. *brassicae* in our tree has to be at least as old as the proposed origin of forams. By looking at our time estimates, it happens that only the two proposals that are based on a time of ~1232 Myr for the origin of rhizaria (i.e., that uses calibration e.1) result in a date for the origin of *P*. *brassicae* that is congruent with the estimated origin of forams (Fig. 3).

**Concordance between different time divergence inferences and earliest forams in the fossil record.**
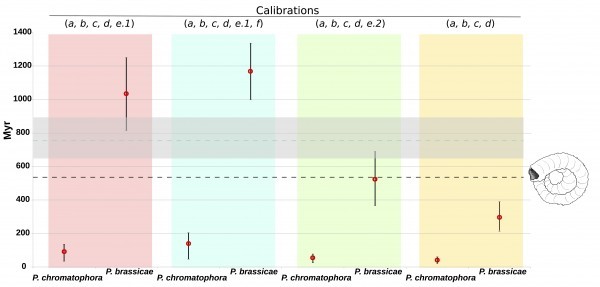
Only calibration schemes assuming a time divergence of rhizaria from stramenopila of ~1232 Myr (e.1) are consistent with the fossil record and proposed time divergence of forams. The horizontal black broken line represents the date of the oldest foram fossil at 545 Myr. The blue broken line represents the estimated origin of forams 770 Myr (650 – 920).

Although the 95% confidence intervals are rater large, the estimates of 93.6 and 141.4 Myr are consistent with the original suggestion that the chromatophore in *P*. *chromatophora* has a minimum age of 60 Myr^6^; and with a more recent proposal of a maximum age of 200 Myr for the divergence of *P*. *chromatophora* from Euglypha rotunda^24^.

If our estimates are correct, the two strains of *P. chromatophora* diverged from each other about (45.7 – 64.7 Myr). The genomes of the chromatophores in these two strains differ in about 33 genes^5^. This give us a rate of ~ 0.25 to 0.36 gene inactivations per million year since the divergence of the two strains. These show that genome reduction in the chromatophore is a process that continues slowly however stately. Whether these genes are been lost by genetic drift or natural selection is still a mater of research. Further refinements on the time of origin of *P*. *chromatophora* and their chromatophores are expected as our understanding of the evolution of rhizaria and the eukaryotic tree of life improves.
